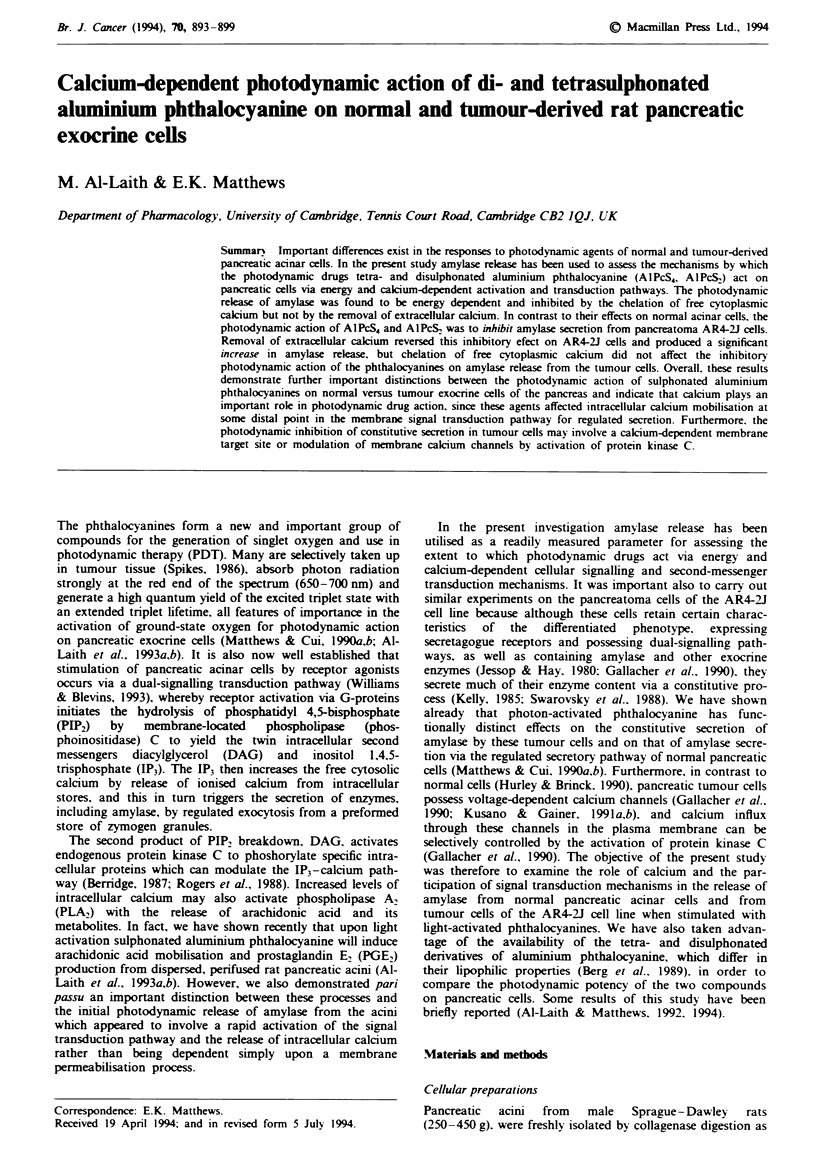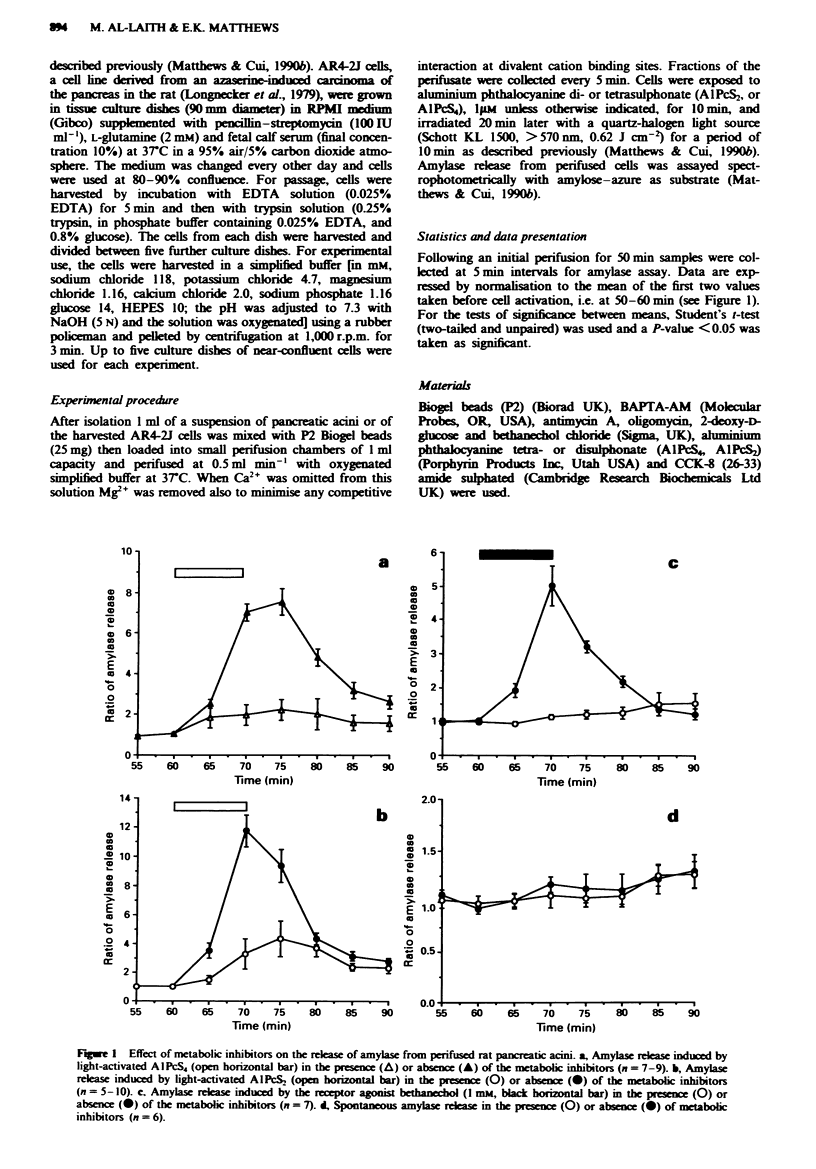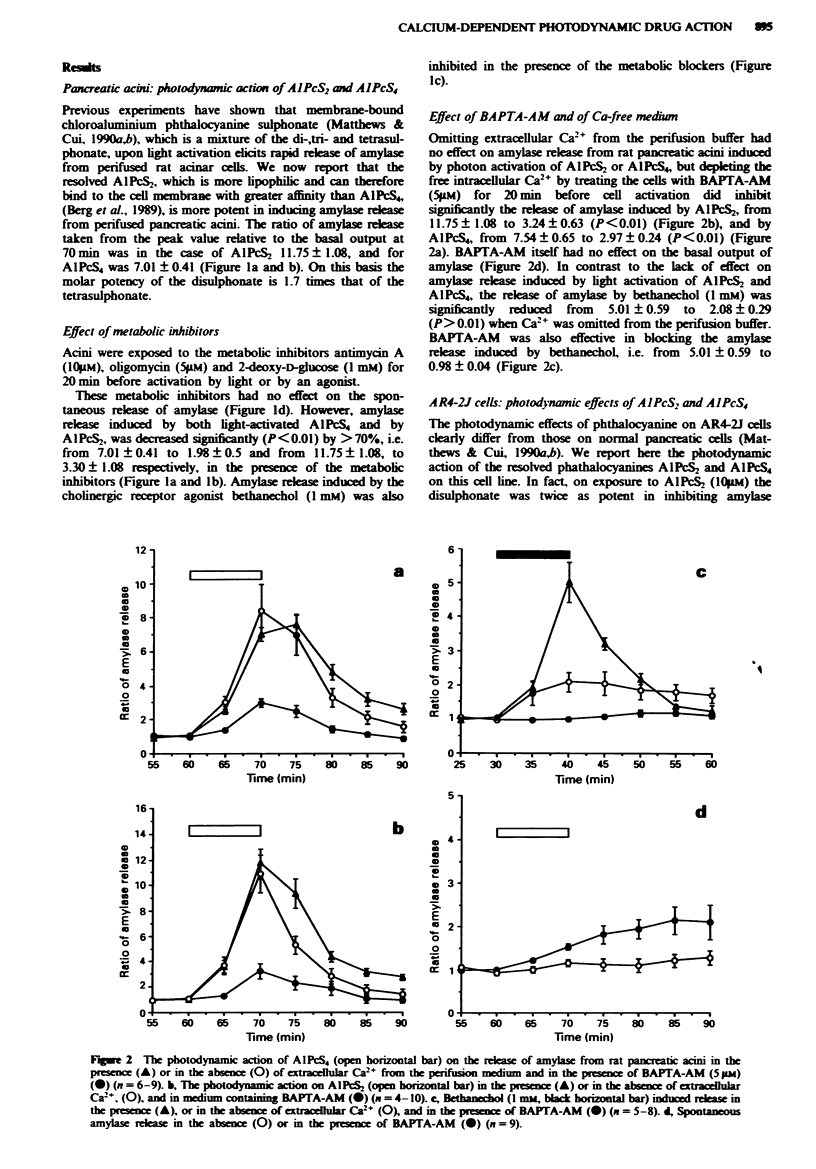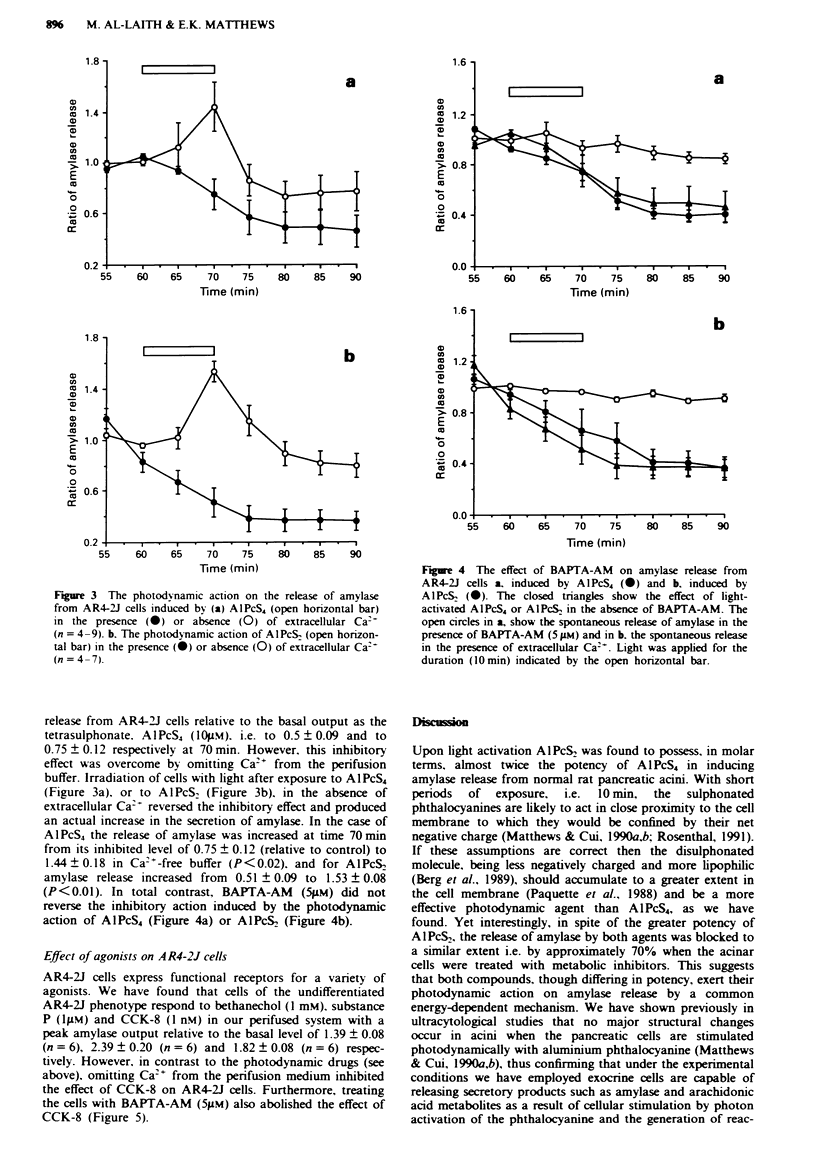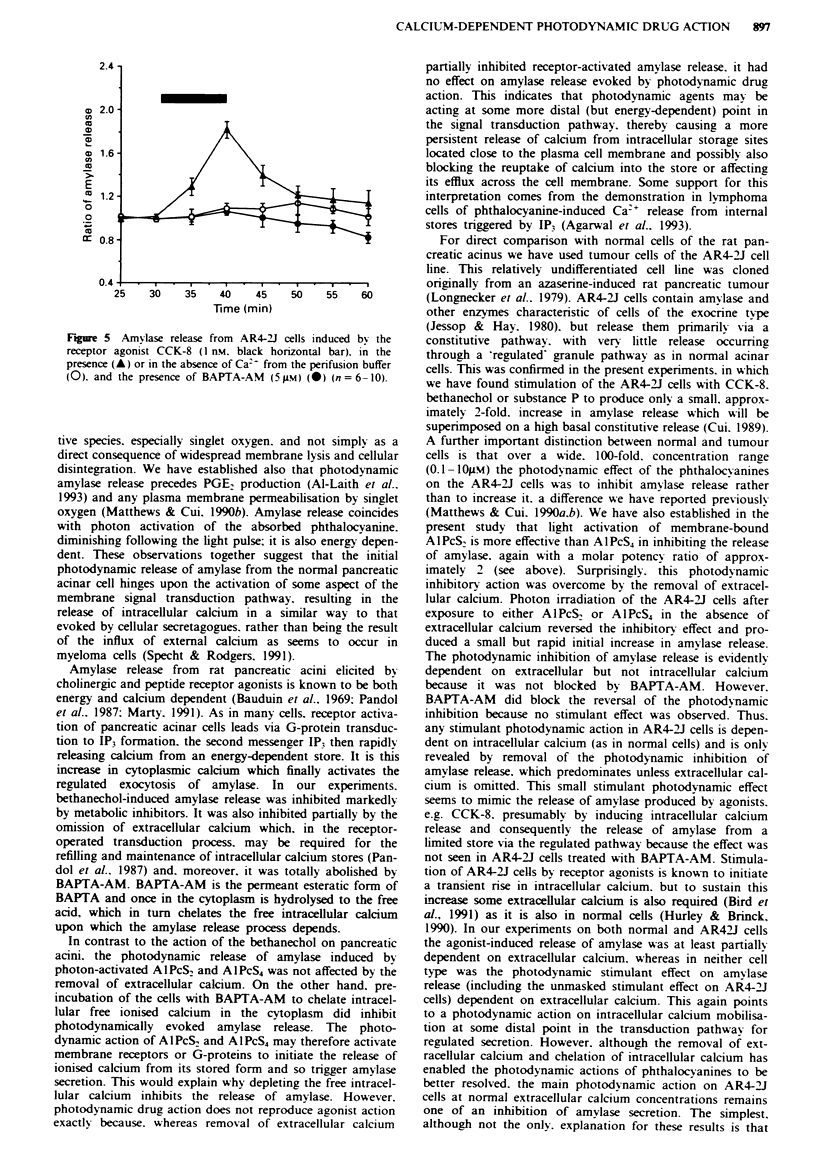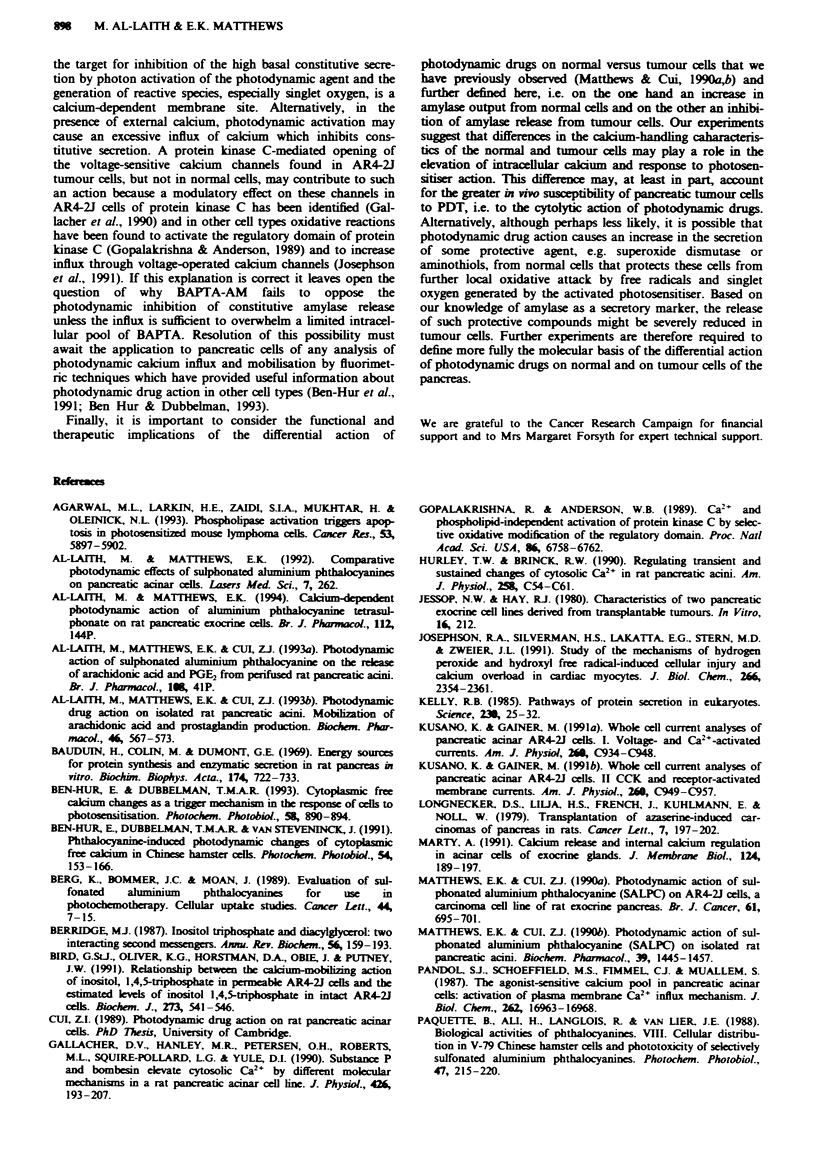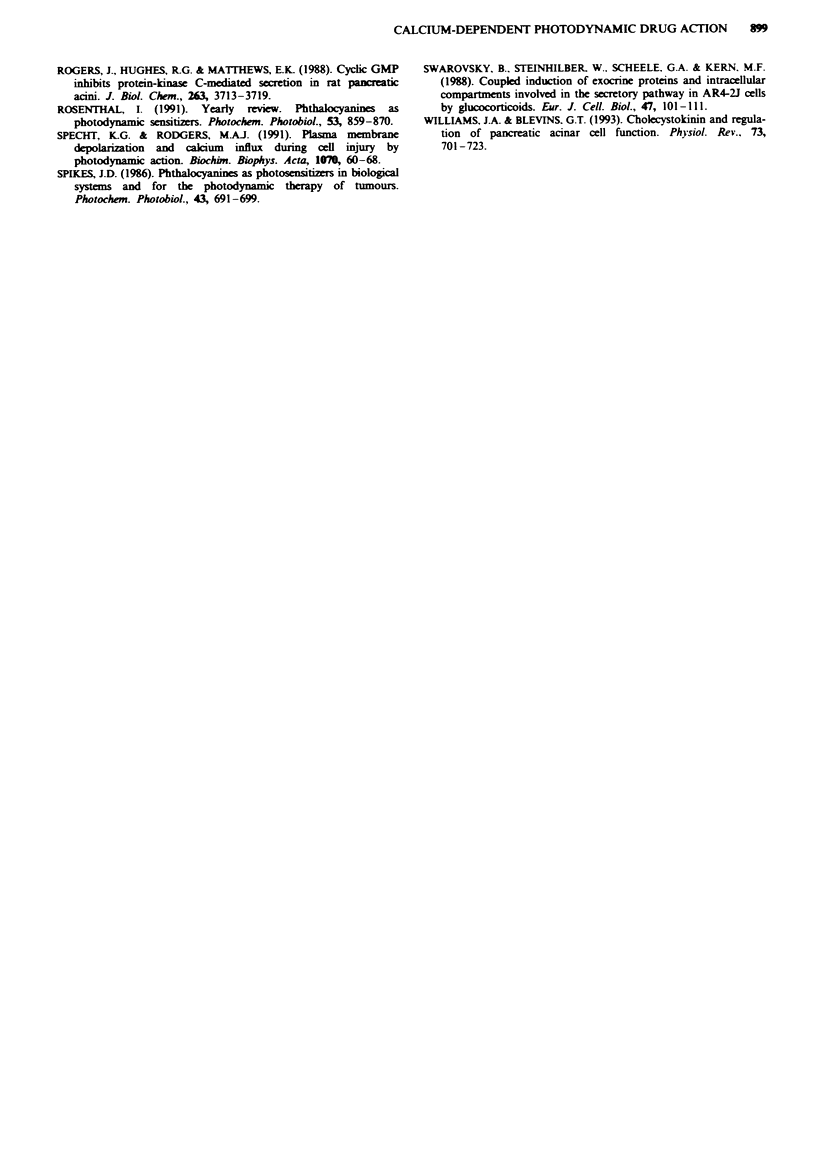# Calcium-dependent photodynamic action of di- and tetrasulphonated aluminium phthalocyanine on normal and tumour-derived rat pancreatic exocrine cells.

**DOI:** 10.1038/bjc.1994.416

**Published:** 1994-11

**Authors:** M. al-Laith, E. K. Matthews

**Affiliations:** Department of Pharmacology, University of Cambridge, UK.

## Abstract

Important differences exist in the responses to photodynamic agents of normal and tumour-derived pancreatic acinar cells. In the present study amylase release has been used to assess the mechanisms by which the photodynamic drugs tetra- and disulphonated aluminium phthalocyanine (A1PcS4, A1PcS2) act on pancreatic cells via energy and calcium-dependent activation and transduction pathways. The photodynamic release of amylase was found to be energy dependent and inhibited by the chelation of free cytoplasmic calcium but not by the removal of extracellular calcium. In contrast to their effects on normal acinar cells, the photodynamic action of A1PcS4 and A1PcS2 was to inhibit amylase secretion from pancreatoma AR4-2J cells. Removal of extracellular calcium reversed this inhibitory effect on AR4-2J cells and produced a significant increase in amylase release, but chelation of free cytoplasmic calcium did not affect the inhibitory photodynamic action of the phthalocyanines on amylase release from the tumour cells. Overall, these results demonstrate further important distinctions between the photodynamic action of sulphonated aluminium phthalocyanines on normal versus tumour exocrine cells of the pancreas and indicate that calcium plays an important role in photodynamic drug action, since these agents affected intracellular calcium mobilisation at some distal point in the membrane signal transduction pathway for regulated secretion. Furthermore, the photodynamic inhibition of constitutive secretion in tumour cells may involve a calcium-dependent membrane target site or modulation of membrane calcium channels by activation of protein kinase C.


Br. J. Cancer (1994), 70, 893-899 © Macmillan Press Ltd., 1994

### Calcium-dependent photodynamic action of di- and tetrasulphonated aluminium phthalocyanine on normal and tumour-derived rat pancreatic exocrine cells

M. Al-Laith & E.K. Matthews

*Department of Pharmacology, University of Cambridge, Tennis Court Road, Cambridge CB2 1QJ, UK*

**Summary** Important differences exist in the responses to photodynamic agents of normal and tumour-derived pancreatic acinar cells. In the present study amylase release has been used to assess the mechanisms by which the photodynamic drugs tetra- and disulphonated aluminium phthalocyanine ($AlPcS_4$, $AlPcS_2$) act on pancreatic cells via energy and calcium-dependent activation and transduction pathways. The photodynamic release of amylase was found to be energy dependent and inhibited by the chelation of free cytoplasmic calcium but not by the removal of extracellular calcium. In contrast to their effects on normal acinar cells, the photodynamic action of $AlPcS_4$ and $AlPcS_2$ was to *inhibit* amylase secretion from pancreatoma AR4-2J cells. Removal of extracellular calcium reversed this inhibitory efect on AR4-2J cells and produced a significant *increase* in amylase release, but chelation of free cytoplasmic calcium did not affect the inhibitory photodynamic action of the phthalocyanines on amylase release from the tumour cells. Overall, these results demonstrate further important distinctions between the photodynamic action of sulphonated aluminium phthalocyanines on normal versus tumour exocrine cells of the pancreas and indicate that calcium plays an important role in photodynamic drug action, since these agents affected intracellular calcium mobilisation at some distal point in the membrane signal transduction pathway for regulated secretion. Furthermore, the photodynamic inhibition of constitutive secretion in tumour cells may involve a calcium-dependent membrane target site or modulation of membrane calcium channels by activation of protein kinase C.

The phthalocyanines form a new and important group of compounds for the generation of singlet oxygen and use in photodynamic therapy (PDT). Many are selectively taken up in tumour tissue (Spikes, 1986), absorb photon radiation strongly at the red end of the spectrum (650-700 nm) and generate a high quantum yield of the excited triplet state with an extended triplet lifetime, all features of importance in the activation of ground-state oxygen for photodynamic action on pancreatic exocrine cells (Matthews & Cui, 1990*a*,*b*; Al-Laith *et al*., 1993*a*,*b*). It is also now well established that stimulation of pancreatic acinar cells by receptor agonists occurs via a dual-signalling transduction pathway (Williams & Blevins, 1993), whereby receptor activation via G-proteins initiates the hydrolysis of phosphatidyl 4,5-bisphosphate ($PIP_2$) by membrane-located phospholipase (phosphoinositidase) C to yield the twin intracellular second messengers diacylglycerol (DAG) and inositol 1,4,5-trisphosphate ($IP_3$). The $IP_3$ then increases the free cytosolic calcium by release of ionised calcium from intracellular stores, and this in turn triggers the secretion of enzymes, including amylase, by regulated exocytosis from a preformed store of zymogen granules.

The second product of $PIP_2$ breakdown, DAG, activates endogenous protein kinase C to phoshorylate specific intracellular proteins which can modulate the $IP_3$-calcium pathway (Berridge, 1987; Rogers *et al*., 1988). Increased levels of intracellular calcium may also activate phospholipase $A_2$ ($PLA_2$) with the release of arachidonic acid and its metabolites. In fact, we have shown recently that upon light activation sulphonated aluminium phthalocyanine will induce arachidonic acid mobilisation and prostaglandin $E_2$ ($PGE_2$) production from dispersed, perifused rat pancreatic acini (Al-Laith *et al*., 1993*a*,*b*). However, we also demonstrated *pari passu* an important distinction between these processes and the initial photodynamic release of amylase from the acini which appeared to involve a rapid activation of the signal transduction pathway and the release of intracellular calcium rather than being dependent simply upon a membrane permeabilisation process.

In the present investigation amylase release has been utilised as a readily measured parameter for assessing the extent to which photodynamic drugs act via energy and calcium-dependent cellular signalling and second-messenger transduction mechanisms. It was important also to carry out similar experiments on the pancreatoma cells of the AR4-2J cell line because although these cells retain certain characteristics of the differentiated phenotype, expressing secretagogue receptors and possessing dual-signalling pathways, as well as containing amylase and other exocrine enzymes (Jessop & Hay, 1980; Gallacher *et al*., 1990), they secrete much of their enzyme content via a constitutive process (Kelly, 1985; Swarovsky *et al*., 1988). We have shown already that photon-activated phthalocyanine has functionally distinct effects on the constitutive secretion of amylase by these tumour cells and on that of amylase secretion via the regulated secretory pathway of normal pancreatic cells (Matthews & Cui, 1990*a*,*b*). Furthermore, in contrast to normal cells (Hurley & Brinck, 1990), pancreatic tumour cells possess voltage-dependent calcium channels (Gallacher *et al*., 1990; Kusano & Gainer, 1991*a*,*b*), and calcium influx through these channels in the plasma membrane can be selectively controlled by the activation of protein kinase C (Gallacher *et al*., 1990). The objective of the present study was therefore to examine the role of calcium and the participation of signal transduction mechanisms in the release of amylase from normal pancreatic acinar cells and from tumour cells of the AR4-2J cell line when stimulated with light-activated phthalocyanines. We have also taken advantage of the availability of the tetra- and disulphonated derivatives of aluminium phthalocyanine, which differ in their lipophilic properties (Berg *et al*., 1989), in order to compare the photodynamic potency of the two compounds on pancreatic cells. Some results of this study have been briefly reported (Al-Laith & Matthews, 1992, 1994).

### Materials and methods

### *Cellular preparations*

Pancreatic acini from male Sprague-Dawley rats (250-450 g), were freshly isolated by collagenase digestion as

Correspondence: E.K. Matthews.
Received 19 April 1994; and in revised form 5 July 1994.

described previously (Matthews & Cui, 1990*b*). AR4-2J cells, a cell line derived from an azaserine-induced carcinoma of the pancreas in the rat (Longnecker *et al*., 1979), were grown in tissue culture dishes (90 mm diameter) in RPMI medium (Gibco) supplemented with pencillin–streptomycin (100 IU $ml^{-1}$), L-glutamine (2 mM) and fetal calf serum (final concentration 10%) at 37°C in a 95% air/5% carbon dioxide atmosphere. The medium was changed every other day and cells were used at 80–90% confluence. For passage, cells were harvested by incubation with EDTA solution (0.025% EDTA) for 5 min and then with trypsin solution (0.25% trypsin, in phosphate buffer containing 0.025% EDTA, and 0.8% glucose). The cells from each dish were harvested and divided between five further culture dishes. For experimental use, the cells were harvested in a simplified buffer [in mM, sodium chloride 118, potassium chloride 4.7, magnesium chloride 1.16, calcium chloride 2.0, sodium phosphate 1.16 glucose 14, HEPES 10; the pH was adjusted to 7.3 with NaOH (5 N) and the solution was oxygenated] using a rubber policeman and pelleted by centrifugation at 1,000 r.p.m. for 3 min. Up to five culture dishes of near-confluent cells were used for each experiment.

### *Experimental procedure*

After isolation 1 ml of a suspension of pancreatic acini or of the harvested AR4-2J cells was mixed with P2 Biogel beads (25 mg) then loaded into small perifusion chambers of 1 ml capacity and perifused at 0.5 ml $min^{-1}$ with oxygenated simplified buffer at 37°C. When $Ca^{2+}$ was omitted from this solution $Mg^{2+}$ was removed also to minimise any competitive interaction at divalent cation binding sites. Fractions of the perifusate were collected every 5 min. Cells were exposed to aluminium phthalocyanine di- or tetrasulphonate ($AlPcS_2$, or $AlPcS_4$), 1μM unless otherwise indicated, for 10 min, and irradiated 20 min later with a quartz-halogen light source (Schott KL 1500, >570 nm, 0.62 J $cm^{-2}$) for a period of 10 min as described previously (Matthews & Cui, 1990*b*). Amylase release from perifused cells was assayed spectrophotometrically with amylose–azure as substrate (Matthews & Cui, 1990*b*).

### *Statistics and data presentation*

Following an initial perifusion for 50 min samples were collected at 5 min intervals for amylase assay. Data are expressed by normalisation to the mean of the first two values taken before cell activation, i.e. at 50–60 min (see Figure 1). For the tests of significance between means, Student's *t*-test (two-tailed and unpaired) was used and a *P*-value <0.05 was taken as significant.

### *Materials*

Biogel beads (P2) (Biorad UK), BAPTA-AM (Molecular Probes, OR, USA), antimycin A, oligomycin, 2-deoxy-D-glucose and bethanechol chloride (Sigma, UK), aluminium phthalocyanine tetra- or disulphonate ($AlPcS_4$, $AlPcS_2$) (Porphyrin Products Inc, Utah USA) and CCK-8 (26-33) amide sulphated (Cambridge Research Biochemicals Ltd UK) were used.

**Figure 1** Effect of metabolic inhibitors on the release of amylase from perifused rat pancreatic acini. **a**, Amylase release induced by light-activated $AlPcS_4$ (open horizontal bar) in the presence (△) or absence (▲) of the metabolic inhibitors (*n* = 7–9). **b**, Amylase release induced by light-activated $AlPcS_2$ (open horizontal bar) in the presence (○) or absence (●) of the metabolic inhibitors (*n* = 5–10). **c**, Amylase release induced by the receptor agonist bethanechol (1 mM, black horizontal bar) in the presence (○) or absence (●) of the metabolic inhibitors (*n* = 7). **d**, Spontaneous amylase release in the presence (○) or absence (●) of metabolic inhibitors (*n* = 6).

### Results

### *Pancreatic acini: photodynamic action of $AlPcS_2$ and $AlPcS_4$*

Previous experiments have shown that membrane-bound chloroaluminium phthalocyanine sulphonate (Matthews & Cui, 1990*a*,*b*), which is a mixture of the di-,tri- and tetrasulphonate, upon light activation elicits rapid release of amylase from perifused rat acinar cells. We now report that the resolved $AlPcS_2$, which is more lipophilic and can therefore bind to the cell membrane with greater affinity than $AlPcS_4$, (Berg *et al.*, 1989), is more potent in inducing amylase release from perifused pancreatic acini. The ratio of amylase release taken from the peak value relative to the basal output at 70 min was in the case of $AlPcS_2$ 11.75 ± 1.08, and for $AlPcS_4$ was 7.01 ± 0.41 (Figure 1a and b). On this basis the molar potency of the disulphonate is 1.7 times that of the tetrasulphonate.

### *Effect of metabolic inhibitors*

Acini were exposed to the metabolic inhibitors antimycin A (10μM), oligomycin (5μM) and 2-deoxy-D-glucose (1 mM) for 20 min before activation by light or by an agonist.

These metabolic inhibitors had no effect on the spontaneous release of amylase (Figure 1d). However, amylase release induced by both light-activated $AlPcS_4$ and by $AlPcS_2$, was decreased significantly ($P < 0.01$) by > 70%, i.e. from 7.01 ± 0.41 to 1.98 ± 0.5 and from 11.75 ± 1.08, to 3.30 ± 1.08 respectively, in the presence of the metabolic inhibitors (Figure 1a and 1b). Amylase release induced by the cholinergic receptor agonist bethanechol (1 mM) was also inhibited in the presence of the metabolic blockers (Figure 1c).

### *Effect of BAPTA-AM and of Ca-free medium*

Omitting extracellular $Ca^{2+}$ from the perifusion buffer had no effect on amylase release from rat pancreatic acini induced by photon activation of $AlPcS_2$ or $AlPcS_4$, but depleting the free intracellular $Ca^{2+}$ by treating the cells with BAPTA-AM (5μM) for 20 min before cell activation did inhibit significantly the release of amylase induced by $AlPcS_2$, from 11.75 ± 1.08 to 3.24 ± 0.63 ($P < 0.01$) (Figure 2b), and by $AlPcS_4$, from 7.54 ± 0.65 to 2.97 ± 0.24 ($P < 0.01$) (Figure 2a). BAPTA-AM itself had no effect on the basal output of amylase (Figure 2d). In contrast to the lack of effect on amylase release induced by light activation of $AlPcS_2$ and $AlPcS_4$, the release of amylase by bethanechol (1 mM) was significantly reduced from 5.01 ± 0.59 to 2.08 ± 0.29 ($P > 0.01$) when $Ca^{2+}$ was omitted from the perifusion buffer. BAPTA-AM was also effective in blocking the amylase release induced by bethanechol, i.e. from 5.01 ± 0.59 to 0.98 ± 0.04 (Figure 2c).

### *AR4-2J cells: photodynamic effects of $AlPcS_2$ and $AlPcS_4$*

The photodynamic effects of phthalocyanine on AR4-2J cells clearly differ from those on normal pancreatic cells (Matthews & Cui, 1990*a*,*b*). We report here the photodynamic action of the resolved phathalocyanines $AlPcS_2$ and $AlPcS_4$ on this cell line. In fact, on exposure to $AlPcS_2$ (10μM) the disulphonate was twice as potent in inhibiting amylase

**Figure 2** The photodynamic action of $AlPcS_4$ (open horizontal bar) on the release of amylase from rat pancreatic acini in the presence (▲) or in the absence (○) of extracellular $Ca^{2+}$ from the perifusion medium and in the presence of BAPTA-AM (5 μM) (●) ($n = 6$–$9$). **b**, The photodynamic action on $AlPcS_2$ (open horizontal bar) in the presence (▲) or in the absence of extracellular $Ca^{2+}$, (○), and in medium containing BAPTA-AM (●) ($n = 4$–$10$). **c**, Bethanechol (1 mM, black horizontal bar) induced release in the presence (▲), or in the absence of extracellular $Ca^{2+}$ (○), and in the presence of BAPTA-AM (●) ($n = 5$–$8$). **d**, Spontaneous amylase release in the absence (○) or in the presence of BAPTA-AM (●) ($n = 9$).

**Figure 3** The photodynamic action on the release of amylase from AR4-2J cells induced by (**a**) $AlPcS_4$ (open horizontal bar) in the presence (●) or absence (○) of extracellular $Ca^{2+}$ ($n = 4-9$). **b**, The photodynamic action of $AlPcS_2$ (open horizontal bar) in the presence (●) or absence (○) of extracellular $Ca^{2+}$ ($n = 4-7$).

**Figure 4** The effect of BAPTA-AM on amylase release from AR4-2J cells **a**, induced by $AlPcS_4$ (●) and **b**, induced by $AlPcS_2$ (●). The closed triangles show the effect of light-activated $AlPcS_4$ or $AlPcS_2$ in the absence of BAPTA-AM. The open circles in **a**, show the spontaneous release of amylase in the presence of BAPTA-AM (5 μM) and in **b**, the spontaneous release in the presence of extracellular $Ca^{2+}$. Light was applied for the duration (10 min) indicated by the open horizontal bar.

release from AR4-2J cells relative to the basal output as the tetrasulphonate, $AlPcS_4$ (10μM), i.e. to $0.5 \pm 0.09$ and to $0.75 \pm 0.12$ respectively at 70 min. However, this inhibitory effect was overcome by omitting $Ca^{2+}$ from the perifusion buffer. Irradiation of cells with light after exposure to $AlPcS_4$ (Figure 3a), or to $AlPcS_2$ (Figure 3b), in the absence of extracellular $Ca^{2+}$ reversed the inhibitory effect and produced an actual increase in the secretion of amylase. In the case of $AlPcS_4$ the release of amylase was increased at time 70 min from its inhibited level of $0.75 \pm 0.12$ (relative to control) to $1.44 \pm 0.18$ in $Ca^{2+}$-free buffer ($P < 0.02$), and for $AlPcS_2$ amylase release increased from $0.51 \pm 0.09$ to $1.53 \pm 0.08$ ($P < 0.01$). In total contrast, BAPTA-AM (5μM) did not reverse the inhibitory action induced by the photodynamic action of $AlPcS_4$ (Figure 4a) or $AlPcS_2$ (Figure 4b).

### *Effect of agonists on AR4-2J cells*

AR4-2J cells express functional receptors for a variety of agonists. We have found that cells of the undifferentiated AR4-2J phenotype respond to bethanechol (1 mM), substance P (1μM) and CCK-8 (1 nM) in our perifused system with a peak amylase output relative to the basal level of $1.39 \pm 0.08$ ($n = 6$), $2.39 \pm 0.20$ ($n = 6$) and $1.82 \pm 0.08$ ($n = 6$) respectively. However, in contrast to the photodynamic drugs (see above), omitting $Ca^{2+}$ from the perifusion medium inhibited the effect of CCK-8 on AR4-2J cells. Furthermore, treating the cells with BAPTA-AM (5μM) also abolished the effect of CCK-8 (Figure 5).

### Discussion

Upon light activation $AlPcS_2$ was found to possess, in molar terms, almost twice the potency of $AlPcS_4$ in inducing amylase release from normal rat pancreatic acini. With short periods of exposure, i.e. 10 min, the sulphonated phthalocyanines are likely to act in close proximity to the cell membrane to which they would be confined by their net negative charge (Matthews & Cui, 1990*a*,*b*; Rosenthal, 1991). If these assumptions are correct then the disulphonated molecule, being less negatively charged and more lipophilic (Berg *et al.*, 1989), should accumulate to a greater extent in the cell membrane (Paquette *et al.*, 1988) and be a more effective photodynamic agent than $AlPcS_4$, as we have found. Yet interestingly, in spite of the greater potency of $AlPcS_2$, the release of amylase by both agents was blocked to a similar extent i.e. by approximately 70% when the acinar cells were treated with metabolic inhibitors. This suggests that both compounds, though differing in potency, exert their photodynamic action on amylase release by a common energy-dependent mechanism. We have shown previously in ultracytological studies that no major structural changes occur in acini when the pancreatic cells are stimulated photodynamically with aluminium phthalocyanine (Matthews & Cui, 1990*a*,*b*), thus confirming that under the experimental conditions we have employed exocrine cells are capable of releasing secretory products such as amylase and arachidonic acid metabolites as a result of cellular stimulation by photon activation of the phthalocyanine and the generation of reac-

**Figure 5** Amylase release from AR4-2J cells induced by the receptor agonist CCK-8 (1 nM, black horizontal bar), in the presence (▲) or in the absence of $Ca^{2+}$ from the perifusion buffer (○), and the presence of BAPTA-AM (5 μM) (●) ($n = 6-10$).

tive species, especially singlet oxygen, and not simply as a direct consequence of widespread membrane lysis and cellular disintegration. We have established also that photodynamic amylase release precedes $PGE_2$ production (Al-Laith *et al.*, 1993) and any plasma membrane permeabilisation by singlet oxygen (Matthews & Cui, 1990*b*). Amylase release coincides with photon activation of the absorbed phthalocyanine, diminishing following the light pulse; it is also energy dependent. These observations together suggest that the initial photodynamic release of amylase from the normal pancreatic acinar cell hinges upon the activation of some aspect of the membrane signal transduction pathway, resulting in the release of intracellular calcium in a similar way to that evoked by cellular secretagogues, rather than being the result of the influx of external calcium as seems to occur in myeloma cells (Specht & Rodgers, 1991).

Amylase release from rat pancreatic acini elicited by cholinergic and peptide receptor agonists is known to be both energy and calcium dependent (Bauduin *et al.*, 1969; Pandol *et al.*, 1987; Marty, 1991). As in many cells, receptor activation of pancreatic acinar cells leads via G-protein transduction to $IP_3$ formation, the second messenger $IP_3$ then rapidly releasing calcium from an energy-dependent store. It is this increase in cytoplasmic calcium which finally activates the regulated exocytosis of amylase. In our experiments, bethanechol-induced amylase release was inhibited markedly by metabolic inhibitors. It was also inhibited partially by the omission of extracellular calcium which, in the receptor-operated transduction process, may be required for the refilling and maintenance of intracellular calcium stores (Pandol *et al.*, 1987) and, moreover, it was totally abolished by BAPTA-AM. BAPTA-AM is the permeant esteratic form of BAPTA and once in the cytoplasm is hydrolysed to the free acid, which in turn chelates the free intracellular calcium upon which the amylase release process depends.

In contrast to the action of the bethanechol on pancreatic acini, the photodynamic release of amylase induced by photon-activated $AlPcS_2$ and $AlPcS_4$ was not affected by the removal of extracellular calcium. On the other hand, preincubation of the cells with BAPTA-AM to chelate intracellular free ionised calcium in the cytoplasm did inhibit photodynamically evoked amylase release. The photodynamic action of $AlPcS_2$ and $AlPcS_4$ may therefore activate membrane receptors or G-proteins to initiate the release of ionised calcium from its stored form and so trigger amylase secretion. This would explain why depleting the free intracellular calcium inhibits the release of amylase. However, photodynamic drug action does not reproduce agonist action exactly because, whereas removal of extracellular calcium partially inhibited receptor-activated amylase release, it had no effect on amylase release evoked by photodynamic drug action. This indicates that photodynamic agents may be acting at some more distal (but energy-dependent) point in the signal transduction pathway, thereby causing a more persistent release of calcium from intracellular storage sites located close to the plasma cell membrane and possibly also blocking the reuptake of calcium into the store or affecting its efflux across the cell membrane. Some support for this interpretation comes from the demonstration in lymphoma cells of phthalocyanine-induced $Ca^{2+}$ release from internal stores triggered by $IP_3$ (Agarwal *et al.*, 1993).

For direct comparison with normal cells of the rat pancreatic acinus we have used tumour cells of the AR4-2J cell line. This relatively undifferentiated cell line was cloned originally from an azaserine-induced rat pancreatic tumour (Longnecker *et al.*, 1979). AR4-2J cells contain amylase and other enzymes characteristic of cells of the exocrine type (Jessop & Hay, 1980), but release them primarily via a constitutive pathway, with very little release occurring through a 'regulated' granule pathway as in normal acinar cells. This was confirmed in the present experiments, in which we have found stimulation of the AR4-2J cells with CCK-8, bethanechol or substance P to produce only a small, approximately 2-fold, increase in amylase release which will be superimposed on a high basal constitutive release (Cui, 1989). A further important distinction between normal and tumour cells is that over a wide, 100-fold, concentration range (0.1–10μM) the photodynamic effect of the phthalocyanines on the AR4-2J cells was to inhibit amylase release rather than to increase it, a difference we have reported previously (Matthews & Cui, 1990*a*,*b*). We have also established in the present study that light activation of membrane-bound $AlPcS_2$ is more effective than $AlPcS_4$ in inhibiting the release of amylase, again with a molar potency ratio of approximately 2 (see above). Surprisingly, this photodynamic inhibitory action was overcome by the removal of extracellular calcium. Photon irradiation of the AR4-2J cells after exposure to either $AlPcS_2$ or $AlPcS_4$ in the absence of extracellular calcium reversed the inhibitory effect and produced a small but rapid initial increase in amylase release. The photodynamic inhibition of amylase release is evidently dependent on extracellular but not intracellular calcium because it was not blocked by BAPTA-AM. However, BAPTA-AM did block the reversal of the photodynamic inhibition because no stimulant effect was observed. Thus, any stimulant photodynamic action in AR4-2J cells is dependent on intracellular calcium (as in normal cells) and is only revealed by removal of the photodynamic inhibition of amylase release, which predominates unless extracellular calcium is omitted. This small stimulant photodynamic effect seems to mimic the release of amylase produced by agonists, e.g. CCK-8, presumably by inducing intracellular calcium release and consequently the release of amylase from a limited store via the regulated pathway because the effect was not seen in AR4-2J cells treated with BAPTA-AM. Stimulation of AR4-2J cells by receptor agonists is known to initiate a transient rise in intracellular calcium, but to sustain this increase some extracellular calcium is also required (Bird *et al.*, 1991) as it is also in normal cells (Hurley & Brinck, 1990). In our experiments on both normal and AR42J cells the agonist-induced release of amylase was at least partially dependent on extracellular calcium, whereas in neither cell type was the photodynamic stimulant effect on amylase release (including the unmasked stimulant effect on AR4-2J cells) dependent on extracellular calcium. This again points to a photodynamic action on intracellular calcium mobilisation at some distal point in the transduction pathway for regulated secretion. However, although the removal of extracellular calcium and chelation of intracellular calcium has enabled the photodynamic actions of phthalocyanines to be better resolved, the main photodynamic action on AR4-2J cells at normal extracellular calcium concentrations remains one of an inhibition of amylase secretion. The simplest, although not the only, explanation for these results is that the target for inhibition of the high basal constitutive secretion by photon activation of the photodynamic agent and the generation of reactive species, especially singlet oxygen, is a calcium-dependent membrane site. Alternatively, in the presence of external calcium, photodynamic activation may cause an excessive influx of calcium which inhibits constitutive secretion. A protein kinase C-mediated opening of the voltage-sensitive calcium channels found in AR4-2J tumour cells, but not in normal cells, may contribute to such an action because a modulatory effect on these channels in AR4-2J cells of protein kinase C has been identified (Gallacher *et al.*, 1990) and in other cell types oxidative reactions have been found to activate the regulatory domain of protein kinase C (Gopalakrishna & Anderson, 1989) and to increase influx through voltage-operated calcium channels (Josephson *et al.*, 1991). If this explanation is correct it leaves open the question of why BAPTA-AM fails to oppose the photodynamic inhibition of constitutive amylase release unless the influx is sufficient to overwhelm a limited intracellular pool of BAPTA. Resolution of this possibility must await the application to pancreatic cells of any analysis of photodynamic calcium influx and mobilisation by fluorimetric techniques which have provided useful information about photodynamic drug action in other cell types (Ben-Hur *et al.*, 1991; Ben Hur & Dubbelman, 1993).

Finally, it is important to consider the functional and therapeutic implications of the differential action of photodynamic drugs on normal versus tumour cells that we have previously observed (Matthews & Cui, 1990*a*,*b*) and further defined here, i.e. on the one hand an increase in amylase output from normal cells and on the other an inhibition of amylase release from tumour cells. Our experiments suggest that differences in the calcium-handling caharacteristics of the normal and tumour cells may play a role in the elevation of intracellular calcium and response to photosensitiser action. This difference may, at least in part, account for the greater *in vivo* susceptibility of pancreatic tumour cells to PDT, i.e. to the cytolytic action of photodynamic drugs. Alternatively, although perhaps less likely, it is possible that photodynamic drug action causes an increase in the secretion of some protective agent, e.g. superoxide dismutase or aminothiols, from normal cells that protects these cells from further local oxidative attack by free radicals and singlet oxygen generated by the activated photosensitiser. Based on our knowledge of amylase as a secretory marker, the release of such protective compounds might be severely reduced in tumour cells. Further experiments are therefore required to define more fully the molecular basis of the differential action of photodynamic drugs on normal and on tumour cells of the pancreas.

We are grateful to the Cancer Research Campaign for financial support and to Mrs Margaret Forsyth for expert technical support.

### References

AGARWAL, M.L., LARKIN, H.E., ZAIDI, S.I.A., MUKHTAR, H. & OLEINICK, N.L. (1993). Phospholipase activation triggers apoptosis in photosensitized mouse lymphoma cells. *Cancer Res.*, **53**, 5897-5902.

AL-LAITH, M. & MATTHEWS, E.K. (1992). Comparative photodynamic effects of sulphonated aluminium phthalocyanines on pancreatic acinar cells. *Lasers Med. Sci.*, **7**, 262.

AL-LAITH, M. & MATTHEWS, E.K. (1994). Calcium-dependent photodynamic action of aluminium phthalocyanine tetrasulphonate on rat pancreatic exocrine cells. *Br. J. Pharmacol.*, **112**, 144P.

AL-LAITH, M., MATTHEWS, E.K. & CUI, Z.J. (1993*a*). Photodynamic action of sulphonated aluminium phthalocyanine on the release of arachidonic acid and $PGE_2$ from perifused rat pancreatic acini. *Br. J. Pharmacol.*, **108**, 41P.

AL-LAITH, M., MATTHEWS, E.K. & CUI, Z.J. (1993*b*). Photodynamic drug action on isolated rat pancreatic acini. Mobilization of arachidonic acid and prostaglandin production. *Biochem. Pharmacol.*, **46**, 567-573.

BAUDUIN, H., COLIN, M. & DUMONT, G.E. (1969). Energy sources for protein synthesis and enzymatic secretion in rat pancreas *in vitro*. *Biochim. Biophys. Acta.*, **174**, 722-733.

BEN-HUR, E. & DUBBELMAN, T.M.A.R. (1993). Cytoplasmic free calcium changes as a trigger mechanism in the response of cells to photosensitisation. *Photochem. Photobiol.*, **58**, 890-894.

BEN-HUR, E., DUBBELMAN, T.M.A.R. & VAN STEVENINCK, J. (1991). Phthalocyanine-induced photodynamic changes of cytoplasmic free calcium in Chinese hamster cells. *Photochem. Photobiol.*, **54**, 153-166.

BERG, K., BOMMER, J.C. & MOAN, J. (1989). Evaluation of sulfonated aluminium phthalocyanines for use in photochemotherapy. Cellular uptake studies. *Cancer Lett.*, **44**, 7-15.

BERRIDGE, M.J. (1987). Inositol triphosphate and diacylglycerol: two interacting second messengers. *Annu. Rev. Biochem.*, **56**, 159-193.

BIRD, G.StJ., OLIVER, K.G., HORSTMAN, D.A., OBIE, J. & PUTNEY, J.W. (1991). Relationship between the calcium-mobilizing action of inositol, 1,4,5-triphosphate in permeable AR4-2J cells and the estimated levels of inositol 1,4,5-triphosphate in intact AR4-2J cells. *Biochem. J.*, **273**, 541-546.

CUI, Z.I. (1989). Photodynamic drug action on rat pancreatic acinar cells. *PhD Thesis*, University of Cambridge.

GALLACHER, D.V., HANLEY, M.R., PETERSEN, O.H., ROBERTS, M.L., SQUIRE-POLLARD, L.G. & YULE, D.I. (1990). Substance P and bombesin elevate cytosolic $Ca^{2+}$ by different molecular mechanisms in a rat pancreatic acinar cell line. *J. Physiol.*, **426**, 193-207.

GOPALAKRISHNA, R. & ANDERSON, W.B. (1989). $Ca^{2+}$ and phospholipid-independent activation of protein kinase C by selective oxidative modification of the regulatory domain. *Proc. Natl Acad. Sci. USA*, **86**, 6758-6762.

HURLEY, T.W. & BRINCK, R.W. (1990). Regulating transient and sustained changes of cytosolic $Ca^{2+}$ in rat pancreatic acini. *Am. J. Physiol.*, **258**, C54-C61.

JESSOP, N.W. & HAY, R.J. (1980). Characteristics of two pancreatic exocrine cell lines derived from transplantable tumours. *In Vitro*, **16**, 212.

JOSEPHSON, R.A., SILVERMAN, H.S., LAKATTA, E.G., STERN, M.D. & ZWEIER, J.L. (1991). Study of the mechanisms of hydrogen peroxide and hydroxyl free radical-induced cellular injury and calcium overload in cardiac myocytes. *J. Biol. Chem.*, **266**, 2354-2361.

KELLY, R.B. (1985). Pathways of protein secretion in eukaryotes. *Science*, **230**, 25-32.

KUSANO, K. & GAINER, M. (1991*a*). Whole cell current analyses of pancreatic acinar AR4-2J cells. I. Voltage- and $Ca^{2+}$-activated currents. *Am. J. Physiol*, **260**, C934-C948.

KUSANO, K. & GAINER, M. (1991*b*). Whole cell current analyses of pancreatic acinar AR4-2J cells. II CCK and receptor-activated membrane currents. *Am. J. Physiol.*, **260**, C949-C957.

LONGNECKER, D.S., LILJA, H.S., FRENCH, J., KUHLMANN, E. & NOLL, W. (1979). Transplantation of azaserine-induced carcinomas of pancreas in rats. *Cancer Lett.*, **7**, 197-202.

MARTY, A. (1991). Calcium release and internal calcium regulation in acinar cells of exocrine glands. *J. Membrane Biol.*, **124**, 189-197.

MATTHEWS, E.K. & CUI, Z.J. (1990*a*). Photodynamic action of sulphonated aluminium phthalocyanine (SALPC) on AR4-2J cells, a carcinoma cell line of rat exocrine pancreas. *Br. J. Cancer*, **61**, 695-701.

MATTHEWS, E.K. & CUI, Z.J. (1990*b*). Photodynamic action of sulphonated aluminium phthalocyanine (SALPC) on isolated rat pancreatic acini. *Biochem. Pharmacol.*, **39**, 1445-1457.

PANDOL, S.J., SCHOEFFIELD, M.S., FIMMEL, C.J. & MUALLEM, S. (1987). The agonist-sensitive calcium pool in pancreatic acinar cells: activation of plasma membrane $Ca^{2+}$ influx mechanism. *J. Biol. Chem.*, **262**, 16963-16968.

PAQUETTE, B., ALI, H., LANGLOIS, R. & VAN LIER, J.E. (1988). Biological activities of phthalocyanines. VIII. Cellular distribution in V-79 Chinese hamster cells and phototoxicity of selectively sulfonated aluminium phthalocyanines. *Photochem. Photobiol.*, **47**, 215-220.

ROGERS, J., HUGHES, R.G. & MATTHEWS, E.K. (1988). Cyclic GMP inhibits protein-kinase C-mediated secretion in rat pancreatic acini. *J. Biol. Chem.*, **263**, 3713–3719.

ROSENTHAL, I. (1991). Yearly review. Phthalocyanines as photodynamic sensitizers. *Photochem. Photobiol.*, **53**, 859–870.

SPECHT, K.G. & RODGERS, M.A.J. (1991). Plasma membrane depolarization and calcium influx during cell injury by photodynamic action. *Biochim. Biophys. Acta*, **1070**, 60–68.

SPIKES, J.D. (1986). Phthalocyanines as photosensitizers in biological systems and for the photodynamic therapy of tumours. *Photochem. Photobiol.*, **43**, 691–699.

SWAROVSKY, B., STEINHILBER, W., SCHEELE, G.A. & KERN, M.F. (1988). Coupled induction of exocrine proteins and intracellular compartments involved in the secretory pathway in AR4-2J cells by glucocorticoids. *Eur. J. Cell. Biol.*, **47**, 101–111.

WILLIAMS, J.A. & BLEVINS, G.T. (1993). Cholecystokinin and regulation of pancreatic acinar cell function. *Physiol. Rev.*, **73**, 701–723.